# Molecular phylodynamics and protein modeling of infectious salmon anemia virus (ISAV)

**DOI:** 10.1186/1471-2148-11-349

**Published:** 2011-12-02

**Authors:** Eduardo Castro-Nallar, Marcelo Cortez-San Martín, Carolina Mascayano, Cristian Molina, Keith A Crandall

**Affiliations:** 1 Department of Biology, 401 Widtsoe Building, Brigham Young University, Provo, UT 84602-5181, USA; 2 Centro de Biotecnología Acuícola, Laboratorio de Virología, Facultad de Química y Biología, Universidad de Santiago de Chile, Avenida Libertador Bernardo O'Higgins 3363 Santiago, Chile; 3 Facultad de Química y Biología, Universidad de Santiago de Chile, Avenida Libertador Bernardo O'Higgins 3363 Santiago, Chile

## Abstract

**Background:**

ISAV is a member of the *Orthomyxoviridae *family that affects salmonids with disastrous results. It was first detected in 1984 in Norway and from then on it has been reported in Canada, United States, Scotland and the Faroe Islands. Recently, an outbreak was recorded in Chile with negative consequences for the local fishing industry. However, few studies have examined available data to test hypotheses associated with the phylogeographic partitioning of the infecting viral population, the population dynamics, or the evolutionary rates and demographic history of ISAV. To explore these issues, we collected relevant sequences of genes coding for both surface proteins from Chile, Canada, and Norway. We addressed questions regarding their phylogenetic relationships, evolutionary rates, and demographic history using modern phylogenetic methods.

**Results:**

A recombination breakpoint was consistently detected in the Hemagglutinin-Esterase (*he*) gene at either side of the Highly Polymorphic Region (HPR), whereas no recombination breakpoints were detected in Fusion protein (*f*) gene. Evolutionary relationships of ISAV revealed the 2007 Chilean outbreak group as a monophyletic clade for *f *that has a sister relationship to the Norwegian isolates. Their tMRCA is consistent with epidemiological data and demographic history was successfully recovered showing a profound bottleneck with further population expansion. Finally, selection analyses detected ongoing diversifying selection in *f *and *he *codons associated with protease processing and the HPR region, respectively.

**Conclusions:**

Our results are consistent with the Norwegian origin hypothesis for the Chilean outbreak clade. In particular, ISAV HPR0 genotype is not the ancestor of all ISAV strains, although SK779/06 (HPR0) shares a common ancestor with the Chilean outbreak clade. Our analyses suggest that ISAV shows hallmarks typical of RNA viruses that can be exploited in epidemiological and surveillance settings. In addition, we hypothesized that genetic diversity of the HPR region is governed by recombination, probably due to template switching and that novel fusion gene proteolytic sites confer a selective advantage for the isolates that carry them. Additionally, protein modeling allowed us to relate the results of phylogenetic studies with the predicted structures. This study demonstrates that phylogenetic methods are important tools to predict future outbreaks of ISAV and other salmon pathogens.

## Background

Infectious salmon anemia virus (ISAV) is a pathogen that has been associated with high fish mortality in the aquaculture industry [1]. The cumulative mortality associated with each outbreak of ISAV in Norway and other countries is very high, reaching up to 100% of fish stock in some cases [2-6]. ISAV has been classified as the only member of Isavirus genus belonging to the *Orthomyxoviridae *family, which includes Influenza viruses [7]. Virions consist mainly of a membranous envelope with two surface glycoproteins and a matrix protein surrounding a ribonucleoprotein complex. Association of nucleoprotein, three polymerase subunits and the genomic RNA, in turn, forms the ribonucleoprotein complex. Virion morphology varies from spherical to pleiomorphic. The enveloped virus particles are of 45-140 nm in diameter, however, highly pleiomorphic particles up to 700 nm in the longest dimension are occasionally observed. The genome organization is consistent with other members of the *Orthomyxoviridae*; comprising of 8 segments of single stranded negative sense RNA ((-) ssRNA), in which each segment encodes one protein except segments 7 and 8, which encode two (European strains)/three (Canadian strains) and two proteins, respectively [8]. Four major structural proteins have been identified, including a 68 kDa nucleoprotein, a 22 kDa matrix protein, a 42 kDa surface glycoprotein named haemagglutinin-esterase protein with receptor-binding and receptor-destroying activity, and a 50 kDa surface glycoprotein with fusion activity, coded by genome segments 3, 8, 6, and 5, respectively. Segments 1, 2, and 4 encode the putative viral polymerase subunits PB1, PB2, and PA. The ORF1 of segment 7 encodes a nonstructural protein with interferon antagonistic properties, while ORF2 encodes a nuclear export protein. The smaller ORF1 of segment 8 encodes the matrix protein, while the larger ORF2 encodes an RNA-binding structural protein also with interferon antagonistic properties [4,9-11].

Regarding the genes coding for surface proteins, *he *genes exhibit a length polymorphism close to the 3' end called highly polymorphic region (HPR) that has been related to virulence, although this is not entirely clear [12-14]. In turn, *f *genes seem to be more conserved in length, with the exception of the region next to the putative protease-processing site (PPPS) [15,16]. The fusion protein in ISAV is synthesized as a precursor protein designated as F0. In order to exert its biological function, F0 must be cleaved by cellular proteases to generate F1 and F2 [17]. Several insertions of 10-12 amino acids (IN1-4), identical to sequences from other genomic segments, have been suggested to confer novel protease cleavage sites [15], and thus they could be subjected to positive selection.

Unlike other members of the *Orthomyxoviridae *family that commonly infect mammals or birds, ISAV naturally infects fishes of the *Salmonidae *family. Although natural outbreaks of ISA have only been recorded in farmed Atlantic salmon (*Salmo salar*), the virus has been isolated also from rainbow trout (*Oncorhynchus mykiss*) [3] and Coho salmon (*Oncorhynchus kisutch*) in Chile [18]. The disease was first reported in Norway and the virus was associated with the disease in 1984. At present, ISAV has been reported in Canada (New Brunswick in 1996 and Nova Scotia in 2000), USA (Maine in 2001), Scotland (1998), The Faroe Islands (2000; Denmark), and recently in Chile [6,19,20]. Because of the worldwide distribution and the potential for serious economical losses, ISAV has been listed as a non-exotic disease for the European Union (EU) and is therefore monitored closely by the European Community Reference Laboratory for Fish Diseases http://www.crl-fish.eu/.

In general, phylogenetic studies of infectious diseases have been centered on human and zoonotic diseases with little attention to enzootic pathogens, although with few exceptions, for example, in plant viruses' crop-related diseases [21-24]. Given the human health impact, phylogenetic studies of *Orthomyxoviruses *have been largely devoted to influenza viruses in particular to influenza A [25,26]. To date, questions related to global distributions, molecular evolution and adaptations, sources of genetic diversity and emergence of new isolates have been extensively addressed in the influenza literature. However, little has been done with its relatives, e.g., ISAV, even when the same genetic structure, and presumably, similar evolutionary forces might be acting on ISAV populations.

In 1999 an ISAV belonging to the North American (NA) genotype was isolated from Coho salmon in Chile and the first outbreak of ISAV in marine-farmed Atlantic salmon in the Southern hemisphere occurred in the same country starting in June 2007. Given that there are no known natural hosts for ISAV in Chile, only a few studies have investigated whether the outbreak source came from North America or Europe by using basic phylogenetic methods [6,15,19,20].

In this study, we apply more sophisticated phylogenetic and population genetic methods to currently available sequence data to address questions regarding the 2007 Chilean ISAV outbreak. In particular, we asked (i) whether recombination is a relevant force acting on *f *and *he *genes, (ii) whether the age estimates of the Chilean clade agree with epidemiological data, and (iii) whether population processes leave marks in ISAV genomes that allow us to reconstruct its demographic history. Finally, we developed new improved structural homology models with which positively selected sites were mapped to relate them with functional traits.

## Results and discussion

We included 70 isolates from Canada, Chile and Norway for which both genes, *f *and *he*, were available along with their sampling dates. The length of the final alignments was 1365 and 1269 bp, respectively (additional file 1). We selected TVM as the best-fit nucleotide substitution model for both genes based on Akaike Information Criterion (AIC) [27]. Rate heterogeneity was estimated as the gamma distributed shape parameter *α *= 0.5740 and 0.6570 for *f and he*, respectively. We also tested for whether the data were best explained by a strict or relaxed molecular clock. Bayes Factor hypothesis testing favored the relaxed model in both cases (10.44 and 21.82 log BF for *f and he*, respectively). This was further supported by the Coefficient of Variation and ucld.stdv parameters.

### Genetic diversity and recombination

In order to explore the data and to see whether recombination plays a recognizable role in ISAV evolution, we estimated the population genetic parameter Theta (*θ*) and recombination rate (*r*) for each population. When calculated under the infinite-sites assumption, the Canadian and Chilean populations were more diverse than the older Norwegian population (0.08579, 0.06247 and 0.01809, respectively). In turn, when using a coalescent sampler estimator, the Norwegian population turned out to be, as expected, more than three times more diverse 0.04774 (95% CI 0.0198-0.08448) than the young Chilean population 0.0142 (95% CI 0.008368-0.020745), and than the Canadian population 0.03523 (95% CI 0.021083-0.051501) as well. Although at first this seems contradictory, coalescent-based estimators of genetic diversity are far more reliable than those based on segregating sites [28]. By taking into account evolutionary history, coalescent-based estimates are more robust to model assumptions and allow us to determine factors influencing the observed patterns (see below). This overall result has practical implications as less diverse populations, i.e., genetically homogeneous, would be more susceptible to vaccines and antiviral treatments whereas more structured populations, i.e., individuals genetically similar within populations but different among populations, make effective vaccine and antiviral deliveries more difficult. So, population genetic inferences can be instrumental in fish health surveillance programs as demonstrated in human health settings [29-32].

**Figure 1:**
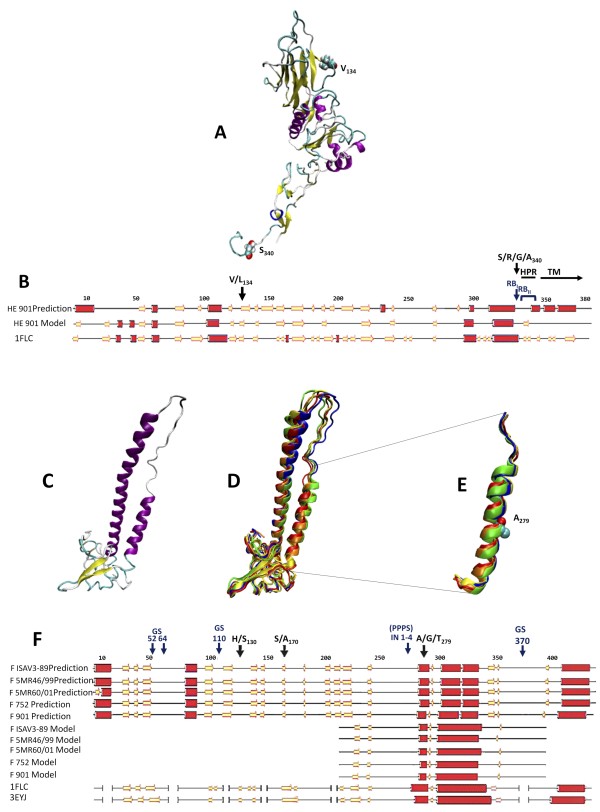
**Schematic representation mapping of positively selected sites and tertiary structure models for both ISAV surface proteins**. **(A) **Structural model of the HE protein from ISAV by homology with the HEF protein from influenza C virus (PDB ID 1FLC) with V134 and S340 residues in Van der Waals representation. **(B) **Alignment of secondary structural prediction (using PSIPRED) for sequence and model of ISAV HE against HEF protein. The alpha helix structural prediction is shown as red cylinders and Beta strands in yellow arrow representation. **(C) **Structural model of the Fusion protein from ISAV 901 (without insertion) by homology with HEF protein from influenza C virus (PDB ID 1FLC) and HE from Influenza A virus (PDB ID 3EYJ). **(D) **Superposition of models of the ISAV fusion protein with and without different insertions. **(E) **Zoomed image of alpha helix, which contains putative fusion peptides and different insertions with A279 residue in Van der Waals representation. **(F) **Alignment of secondary structural prediction (using PSIPRED) for different sequences and models of ISAV Fusions against HEF protein. The alpha helix structure prediction is shown as red cylinders and Beta strands as yellow arrows. Positively selected sites were deemed as such when the dN/dS rate ratio was greater than 1 and with a *p*-value less than 0.1. Amino acid numbering according to ISAV752 09 (ADF36500 for Hemagglutinin and ADF36499 for Fusion). Black arrows denote positively selected sites. Blue labels indicate landmarks in the genes, i.e., GS, glycosylation site; IN, insertion; RB, recombination breakpoint; PPPS, putative protease-processing site; HPR, Highly Polymorphic Region; TM, Transmembrane domain.

Antisense (-) ssRNA viruses are less prone to recombination than their (+) sense counterparts [33,34]. In principle this would arise from the impossibility of (-) ssRNA viruses to serve as templates for negative sense strand synthesis once the genome is uncoated, in turn rendering copy choice recombination less likely. In addition, (-) ssRNA genomes are generally protected by viral nucleoproteins that prevent genome exposure to the cytoplasm (and cell machinery) and to come in close contact with other genome copies, thus rendering copy-choice recombination also unlikely. Also, the segmented genome structure of some (-) ssRNA relaxes even more the occurrence of recombination by yielding the same outcome when shuffling segments (reassortment). In agreement with this hypothesis, our results showed a low overall recombination force (*r*) of (1.173 × 10^-3 ^(1.90 × 10^-4 ^- 3.50 × 10^-3^)) expressed as the ratio of recombination rate per site over substitution rate per site. However, recombination does happen in human [35-37] and avian [38] strains of influenza viruses as well as in some segments in ISAV, namely segments 6 and 5 [12-14]. In this sense, we tested our dataset to see whether or not it is possible to detect recombination breakpoints. Using a suite of methods [39], we consistently detected one possible recombination breakpoint in the vicinity of the *he *HPR region. This result was confirmed by substitution and phylogenetic algorithms and was further corroborated by phylogenetic topological incongruence using a genetic algorithm (GARD; Figure 1B). Since full-length HPR (HPR0) has been detected only in natural populations (not virulent) and different HPR alleles co-occur in the same fish-farming location, these results support the hypothesis that the HPR region is the product of template switching during replication (unequal recombination), and opens the possibility of testing it in controlled experimental settings. Early indications of recombination in the *he *gene were reported in 2001, when researchers looked for topological incongruence and possible recombination patterns 'by eye' within *he *Norwegian sequences [40]. Interestingly, they found that recombination was associated with the geographic location of the isolates and that it occurred at either side of the HPR region. Our results provide statistical support for this initial observation as we detected a topological incongruence at nucleotide 900 (KH-adjusted *p*-value = 2 × 10-4; Figure 1B). We also detected recombination breakpoint signals at either side of the HPR; however, it was not possible to detect parental sequences with confidence (Rdp, maxchi, chimera and Genconv; Figure 1B). This evidence contradicts the hypothesis of Cunningham et al. [41] that the most plausible mechanism of recombination is that each HPR comes from partial deletions of precursor HPR0.

Regarding the *f *gene, we do not detect any recombination breakpoints. At first this was surprising since small non-homologous fragments have been observed at around 266-276, which might confer novel protease cleavage sites (IN1-4) and therefore have a fitness impact. We think there are at least two potential reasons for our inability to detect recombination in this gene region. First, small fragments are hard to detect with current methods because of the low information they contain. Second, methods are not designed to explicitly look for non-homologous recombination. Also, it would be hard to detect if the INs are evolutionarily linked to the *f *gene and/or have similar substitution rates.

### Phylogenetic inference and past population dynamics

In order to estimate phylogenetic relationships, we used Bayesian phylogenetic methods to test the Chilean outbreak isolates for monophyly, to estimate rates of molecular evolution across the genomes, and estimate divergence times for clades of particular interest (e.g., geographically isolated outbreaks). Recombination has detrimental effects on phylogeny estimation and phylogeny-based analyses [34,42,43] and thus the recombinant portion of the *he *gene was not included in these analyses. Segmented viruses have the potential to re-assort their segments in novel combinations, which in phylogenetic terms has the same effect as recombination. Therefore, we estimated phylogenies and molecular evolution parameters separately for each gene since concatenating both datasets would render inaccurate inferences as ISAV might exhibit reassortment between segments.

**Figure 2:**
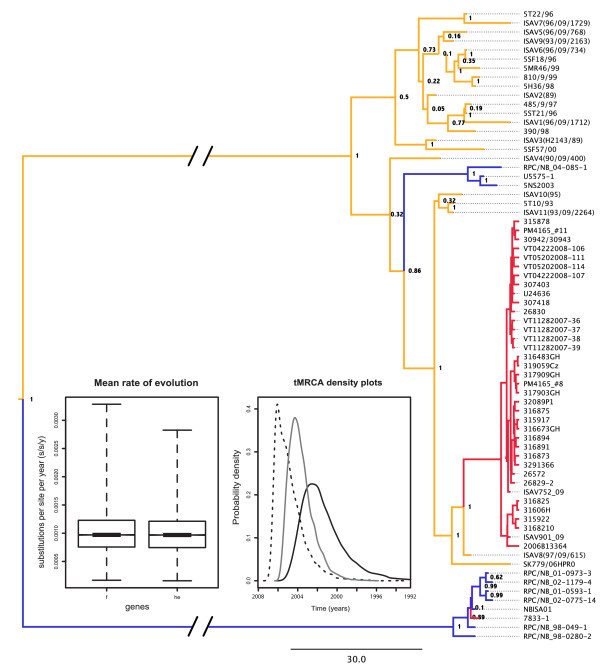
**Bayesian Phylogenetic Inference for ISAV *f *gene**. Maximum clade credibility (MCC) phylogeny depicting the evolutionary relationships of isolates from Europe, Canada and Chile. Colors indicate ancestral locations as inferred from Ancestral State Reconstruction (ASR). Red = Chile, Blue = Canada and Yellow = Europe. Support is expressed as posterior probabilities for major nodes. Inset = boxplots of evolutionary rates for the *f *and *he *genes and density plots for node age estimates (black dashed line = Chilean *he *minor clade; black line = Chilean *he *major clade; grey line = Chilean *f *clade). Isolate NBISA01 belongs to the Canadian clade and it was reported in Chile in 2001.

In the case of the *f *gene, the Chilean outbreak clade was inferred as monophyletic suggesting that ISAV was introduced once in Chilean fish farming (Figure 2). However, the *he *gene tree (additional file 2) shows that outbreak isolates spread in two clades, i.e., paraphyletic; one composed of most of the outbreak isolates and the other composed of three isolates that are also closely related to isolates from Norway (VT11282007-38/-39 and 2006B13364 in additional file 2). For both genes, posterior probability support was close or equal to 1, either for *f *monophyletic outbreak clade or *he *paraphyletic outbreak clades. We speculate that this might be the consequence of reassortment as *he *genes from Chile exhibit two evolutionary pathways. Interestingly, the HPR0 allele (SK779/06HPR0 in Figure 2) is suggested to be the precursor of all HPR alleles by differential deletion of the polymorphic region [12]. However, in both gene trees, SK779/06HPR0 (not virulent) does not share a most recent common ancestor with all the alleles, but it does for the Chilean outbreak alleles. This result suggests that the ancestor of the HPR0 allele is not necessarily the precursor of all modern HPRs. Nevertheless, this result agrees with the hypothesis that the ISAV that finally led to the Chilean outbreak was not virulent [20]. Overall, these results are in rough agreement with previously published phylogenies using other methods. However, our analysis fully resolved relationships in crown nodes and allowed us to estimate molecular evolutionary parameters taking into account phylogenetic and parameter uncertainty. We focused in two events in the evolutionary history of the virus, the time in which Norwegian and Chilean isolates diverged, and the time in which the Chilean epidemic clade expanded. Whether it was in Norway or in Chile, the most recent common ancestor (MRCA) between Norwegian and Chilean isolates was estimated to have occurred in 1995 [1992-1996 95% Highest Probability Density (HPD)] and the Chilean clade expansion in 2003 (2001-2005 95% HPD) for the *f *gene. For the *he *gene, in turn, the MRCA was 1989 (1982-1994 95% HPD) and 2001 (1996-2004 95% HPD). This is in agreement with reported distance-based analyses for the *f *gene but not for the *he *gene [20]. However, those methodologies do not provide confidence estimates in a probabilistic framework, which, in turn, could allow for meaningful comparisons. For instance, our estimates show that both segments have different evolutionary histories since they coalesce at significantly different points back in time (0.83 and 0.94 posterior probabilities for *he *and *f *Chilean clades, respectively). Together with the paraphyly of the *he *outbreak clade, the results suggest that the ancestor between Norwegian and Chilean isolates underwent reassortment of at least both surface protein genes. Ancestral State Reconstruction (ASR) clearly shows that Chilean isolates came from Norway as previously hypothesized and this result is consistent across both genes (color-coded in Figure 2 and additional file 2).

As other (-) ssRNA viruses, ISAV exhibited extremely high substitution rates. Similar to influenza viruses, ISAV replication machinery lacks proofreading activity, which has been proposed as the increased source of variability in RNA viruses [33,44]. Accordingly, substitution rates were estimated at 7.83 × 10^-4 ^substitutions per site per year (s/s/y) (4.31 × 10^-4 ^- 1.18 × 10^-3 ^95% HPD) for the *f *gene and 1.039 × 10^-3 ^(s/s/y) (3.87 × 10^-4 ^- 1.86 × 10^-3 ^95% HPD) for the *he *gene (Figure 2).

**Figure 3:**
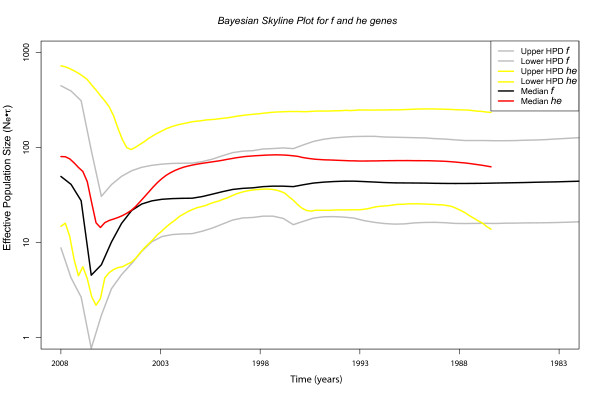
**Bayesian Skyline Plot reconstruction for the *f *and *he *genes**. Black and red lines represent median estimates of effective population size, scaled by generation time in years (τ), for the *f *and *he *genes, respectively. Grey and yellow lines represent 95% Highest Probability Density (HPD) for both genes with grey for *f *and yellow for *he*, respectively.

An interesting aspect of phylogenetic analyses is to infer processes from patterns in the time coordinate. We hypothesized that if ISAV is such a fast-evolving entity, its genome must contain information that could be used to infer ecological and/or population processes. For ISAV, we inferred a population level process of a bottleneck, i.e., a population going through one or more generations of small size followed by subsequent population growth, perhaps associated with the outbreak it recently experienced around 2007 when it was first officially reported in Chile http://www.sernapesca.cl. We reconstructed generation time-scaled effective population size (N_ef_*τ) through time to see whether or not it is possible to recover that bottleneck using the Bayesian Skyline non-parametric model (BSP) [45] (Figure 3). Interestingly, the analysis shows a strong reduction in population size around 2007 followed by a nearly exponential growth. In particular, the greatest reduction in population size was inferred from the *f *gene and occurred in 2006.42 and 2006.02 for the *he *gene (Figure 3). One assumption of analyses based on the coalescent and thus the BSP is that the dataset comprises one non-structured population. Although not explored in depth in the literature, population structure, in principle, could lead to biased estimates of substitution rates and associated parameters. This, in turn, can produce patterns in the BSP that reflect changes in the degree of structure instead of changes in the overall population size, especially when using a single marker [46,47]. To address this potential problem, we calculated Parsimony Score (PS) and Association Index (AI) for both genes under study. These statistics aim to assert the degree of association of a discrete character, geographic location in this case, with a posterior distribution of trees. Both PS and AI statistics were significant (*p*-value < 0.05), suggesting that it is possible to reject the null hypothesis of one non-structured population. Considering that two independent genes showed similar patterns, and that the BSP correlates well with surveillance data, plus the agreement of substitution rate estimates with others [20,48], we think is safe to assume that these results reflect a population bottleneck. Certainly, simulation studies will aid to assert how robust BSP estimates are to structured populations.

Although controversial, Vike et al. [19] suggested that the source of ISAV in the Chilean outbreak were infected eggs brought from Norway, which could be explained by the fact that until 2008 this country was the main provider of embryonated eggs http://www.sernapesca.cl. It is clear that the Chilean outbreak clade has a sister relationship with Norwegian isolates (closely related to ISAV8 (97/09/615); see Figure 2), that the basal clades and ancestral nodes in the phylogeny are from Norway, and that the tMRCA is around 1989-1996, which matches the date when egg importation grew from less than 20 million to 50 million imported units http://www.sernapesca.cl. In addition, the BSP analysis shows that the ISAV population increased exponentially since 2006, suggesting that the virus population remained constant since 2001 (tMRCA of Chilean outbreak clade) and that the outbreak was publicly reported one year after this predicted population expansion. These results constitute valuable information for fish health officials to make informed future decisions based on what contingency measures were implemented at the time of transmission from Norway to Chile. For instance, it is very interesting to note that in 2011 the main ISAV genotype detected in Chile corresponded to HPR0 http://www.sernapesca.cl. Conversely, HPR7b was nearly 80% prevalent between epidemic years 2007 and 2008 [20]. This could be attributed to the change of source material from Norway to Iceland in 2009. For a future study it would be interesting to address the origin of newly detected avirulent HPR0 strains that would come from a country other than Norway.

### Selection analysis and structure models

In order to find potentially relevant sites in the primary sequence of both surface genes and to determine possible structure-function relations, we used a two-rate Fixed-Effects likelihood (two-rate FEL) approach to find positively selected sites (dN/dS rate ratio *>*1). In a gene-based approach, we found that both genes are evolving under purifying selection (dN/dS rate ratio *<*1) as expected for fast-evolving entities with relatively small genomes (0.2038 and 0.1382 for *he and f *genes, respectively).

Using a site-by-site approach, we found that the *he *gene has two sites under positive selection within codons in positions 134 and 340 (Figure 1B; numbering according to ISAV752 isolate). In the *he *gene, amino acid Val/Leu_134 _is located specifically on the globular distal domain (Figure 1A), which is related to HE protein main function, i.e., cell receptor recognition [49]. Amino acid Ser/Arg/Gly/Ala_340 _lies upstream the HPR region, located in the stalk of the protein (Figure 1A), which prompted us to hypothesize that it might be involved in recombination. Interestingly, the expression of recombinant ISAV HEΔ_339-351 _results in decreased expression levels, impaired salmon serum recognition, and hema-adsorption activity. In contrast, HEΔ_349-351 _shows normal parameters [49], suggesting that positively selected amino acid in position 340 is due to its great importance for HE activity.

We also found three sites under positive selection in the *f *gene within codons that code for amino acid residues 130, 170, and 279 (Figure 1F). It was not possible to reliably model the *f *gene protein product structure. However, we were able to map amino acid Ala/Gly/Thr_279_, which lies immediately downstream of insertions (INs) [6]. This constitutes an interesting finding given that insertions in this region have been reported to confer novel PPPS [15], as outlined in Figure 1F. In addition, Ala/Gly/Thr_279 _lies immediately downstream to Arg_278 _(Arg_267 _in ISAV fusion without insertion), a putative cleavage site [17] that has been suggested as a virulence factor [50]. Conversely, a recent study suggested that substitution of Gln/His_266 _to Leu_266 _(two positions upstream to our predicted site) in ISAV fusion protein without insertion is in association with an increase of virulence behavior, as seen in Chilean ISAV 901 strain [13,15]. This has epidemiological implications as isolates carrying these elements would have an advantage compared to those that do not. This finding predicts that the frequency of these alleles will increase in the populations where they are present, e.g., IN4, which is only found in Chile with almost 80% prevalence. It has been suggested that INs close to the cleavage site exposes this region to the solvent probably helping in recognition and processing. Our improved model shows that insertions lie inside the alpha helix structure that is enlarged with subsequent addition of residues, which leads to the consequent exposure of PPPSs (Figure 1D and 1E), in comparison with ISAV901 fusion protein without insertion (Figure 1C). These findings also support the notion that IN's serve as virulence determinants. For instance, the unique insertion, IN4, provides a cleavage site for Asp-N endopeptidase metalloprotease, which has been detected in the skin mucosa of salmonids [15]. Since the HPR in *he *as well as the INs in *f *are associated with virulence and are also under natural selection pressure, we think that experimental confirmation of these results will lead to a better understanding of molecular determinants of pathogenicity for both surface proteins.

## Conclusions

In summary, this study investigated the evolutionary history of ISAV, an important economic infection for salmon culture worldwide. Although less exploited in non-human diseases, phylogenetic and phylodynamic analyses characterize pathogens in space and time, which is perfectly suited for surveillance programs. For instance, identification of the origin of an outbreak could aid officials in formulating management policies. Though not pursued in this study, phylogeographic analyses could help identify virus sources and venues from which pathogens are spreading into different populations or fisheries. Reconstructing past population dynamics could help monitor how an infectious agent is changing or reacting to sanitary interventions in terms of its effective population size. Also, examining molecular sequences for positive selection, all in a structural context, can identify determinants of virulence or other molecular adaptations.

In this study we found that ISAV, as its relatives, (i) exhibit a low recombination rate, though recombination is present in the *he *surface gene. We hypothesized that HPR region diversity is governed by recombination probably due to template switching. (ii) We also provided more evidence in favor of the Norwegian origin hypothesis in a coherent phylogenetic and statistical framework. Contrary to the widely accepted view [41], (ii) ISAV HPR0 genotype is not the precursor of all ISAV *he *genotypes, but appears to share a common ancestor with the Chilean outbreak clade. Finally, (iv) we identified potential determinants of fitness as identified by being under positive selection in a structural context; the protein models allowed us to relate the results of phylogenetic studies with the predicted structures, which suggest an important structural function for the predicted positively selected sites. Our analysis shows that the ISAV outbreak remained undetected in Chile since 2001 and that the population started to increase prior to the identification of the outbreak. Thus, we demonstrate the utility of evolutionary analyses in characterizing temporal, spatial, and mechanistic aspects of ISAV outbreaks, as well as their use in predicting future outbreaks of ISAV and other pathogens. Undoubtedly, collaboration between basic experimental research and phylogenetic fields will help understand the dynamics of ISAV spread in space and time as it has proven fruitful in human health and emerging infectious diseases studies.

## Methods

### Sequences and alignment

For this study, we focused on the two most abundant genes available, which also have been linked to virulence and used extensively for genotyping, namely the Fusion (*f*) and Hemagglutinin-esterase (*he*) genes. Sequences ranging from 1989-2009 were obtained from GenBank according to several criteria. First, in order to implement a molecular clock, we selected only full-length sequences with reported isolation dates. Second, we only used isolates for which both target genes were sequenced. After these criteria were applied, seventy full-length sequences from each gene were retrieved from GenBank (accession numbers in additional file 3) from strains isolated in Canada, Norway, and Chile (alignment in additional file 1). To avoid alignment artifacts and to come up with an optimal statement of homology, nucleotide sequences were visualized in Seaview 4.2.7 [51] and then converted to amino acid sequences for alignment with MAFFT [52] under L-INS-i algorithm, thereby preserving the open reading frame for these protein coding sequences. Amino acid sequences were then back translated into their original nucleotide sequences for further analyses.

### Genetic diversity and recombination

Genetic diversity (θ) within populations was estimated both by the traditional segregating sites approach of Watterson [53] (implemented in DnaSP5 [54]) and by a coalescent Bayesian Markov Chain Monte Carlo method (BMCMC [55]; implemented in Lamarc 2.1.3 [56]). Recombination breakpoint analysis was performed under a suit of methods looking for different types of recombination evidence (Rdp, Genconv, MaxChi, Chimaera as primary methods and Bootscanning and Siscan as secondary (summarized in [57]) as implemented in Rdp3 [39]. Windows and step sizes were varied to refine primary evidence of recombination as suggested in the user manual. In addition, recombination detection was performed on both genes by using a genetic algorithm approach looking at topological incongruence (GARD) [58] as implemented in the http://datamonkey.org[59] server.

### Phylogenetic inference, molecular clock, substitution rates and demographic history

The best-fit model of evolution was selected under Akaike information Criterion [27] (AIC) and parameter values using model averaging as implemented in jModelTest 1.0.1 [60]. The best-fit model was used for subsequent analyses; nevertheless, base frequencies and rates were co-estimated with the phylogeny. A phylogenetic tree was inferred using Bayesian Inference (BI) as optimality criterion as implemented in the Beast 1.6.1 package [61]. Posterior probabilities (PP) were approximated by running four independent analyses of 1 × 10^8 ^steps sampled every 1 × 10^5 ^each. A burn-in period was defined using Tracer [57] by testing for convergence on likelihood scores. Burn-in was defined as the period before convergence and those runs were discarded accordingly. Also, we assessed convergence and mixing by checking the effective sample size statistic (ESS > 500) and the trace itself. Strict molecular clock was rejected on the basis of Bayes Factor hypothesis testing between uncorrelated lognormal clock (H1) and strict clock (H0) as implemented in Tracer [62]. A lognormal prior with a mean centered on the reported Influenza A HA gene substitution rate [26] and tip dates were used to calibrate the clock. The same analysis was run 'on empty' to assess the influence of the selected priors. Estimating Association Index (AI), Parsimony Score (PS), and Maximum Clade number (MC) statistics tested the extent of geographic structure in BaTS [63]. Past population dynamics of both genes were inferred using the Bayesian Skyline Plot (BSP) model as a tree prior [45]. Ancestral State Reconstruction (ASR) was estimated used the method of Lemey et al. [64] implemented in BEAST as described online http://beast.bio.ed.ac.uk/Tutorials#Phylogeography_tutorials

### Selection analyses and structure models

Both genes, *he *and *f*, were investigated for evidence of positive selection. First, a global analysis was performed using a counting method (SLAC). Then, a site-by-site analysis was performed using Fixed-Effects likelihood method as implemented in HyPhy 2.0 [65,66]. Differences in selective pressures between the Chilean clade and the rest of the tree were estimated with SelectionLRT.bf script also within the HyPhy 2.0 package.

To assess whether selected sites lay on structural and functionally relevant regions of proteins, *f *and *he *3D structure models were build using the following protocol. The sequences of the *he *and the *f *genes were obtained from GenBank (ADF36510.1; ABG65799.1; AAX46277.1; AAX46259.1; ADF36509.1 and ADF36499.1). Alignments for HE protein were obtained by modeler 9v9 [67], and for F the server CLUSTALW was used [68]. To verify that the alignments were suitable, we used the secondary structure prediction server PSIPRED http://bioinf.cs.ucl.ac.uk/psipred/[69]. In order to generate the F protein model, we used two templates: hemagglutinin-esterase-fusion glycoprotein structure of influenza C virus (PDB ID: 1FLC) (3.20Å) and hemagglutinin-fusion glycoprotein structure of influenza A virus (PDB ID: 3EYJ) (2.60 Å), with identities of 21% and 20%, respectively. For the HE protein model, we used hemagglutinin-esterase fusion glycoprotein structure of influenza C virus (PDB ID: 1FLC) with 19% identity as template. Finally, both models were minimized (50,000 steps) and a trajectory of 1 ns was obtained by molecular dynamics using the CHARMM force field [70] in NAMD program [71]. The quality of the models was tested by the Anolea server [72].

## Authors' contributions

ECN conceived the study, obtained and analyzed the molecular data and wrote the manuscript. MCS, C. Ma and C. Mo developed protein models, analyzed data and helped writing the manuscript. KAC conceived the study, advised on analyses, outlined, revised and edited the manuscript. All authors agreed with the final version of the manuscript.

## Supplementary Material

Additional file 1 **Nucleotide alignments**. Datasets used in this study. File is formatted as a nexus file, which can be read by most sequence editor software. Taxa names are designated according to isolate name and geographic location. The *f *gene sequence goes from nucleotide 1 to 1368 and *he *gene sequence from 1369 to 2640. A MrBayes block with a character set is provided at the end of the file that can be read by BEAST. Click here for file

Additional file 2 **Hemagglutinin-Esterase Maximum clade credibility (MCC) phylogeny**. Phylogenetic tree representing evolutionary relationships between the *he *genes of selected isolates. Colors represent country of origin and inferred ancestral states as in Figure 2. Click here for file

Additional file 3 **Sequence information and accession numbers**. Excel readable spreadsheet listing isolate names, their country of origin, year of isolation, and their GenBank accession numbers. Click here for file
